# Insula as the Interface Between Body Awareness and Movement: A Neurofeedback-Guided Kinesthetic Motor Imagery Study in Parkinson’s Disease

**DOI:** 10.3389/fnhum.2018.00496

**Published:** 2018-12-07

**Authors:** Sule Tinaz, Kiran Para, Ana Vives-Rodriguez, Valeria Martinez-Kaigi, Keerthana Nalamada, Mine Sezgin, Dustin Scheinost, Michelle Hampson, Elan D. Louis, R. Todd Constable

**Affiliations:** ^1^Division of Movement Disorders, Department of Neurology, Yale School of Medicine, Yale University, New Haven, CT, United States; ^2^Department of Neurology, Faculty of Medicine, Istanbul University, Istanbul, Turkey; ^3^Department of Radiology & Biomedical Imaging, Yale School of Medicine, Yale University, New Haven, CT, United States; ^4^Department of Chronic Disease Epidemiology, Yale School of Public Health, Yale University, New Haven, CT, United States; ^5^Center for Neuroepidemiology and Clinical Neurological Research, Yale School of Medicine, Yale University, New Haven, CT, United States

**Keywords:** functional magnetic resonance imaging, functional connectivity, interoception, intention, basal ganglia, dorsomedial frontal cortex

## Abstract

Intentional movement is an internally driven process that requires the integration of motivational and sensory cues with motor preparedness. In addition to the motor cortical-basal ganglia circuits, the limbic circuits are also involved in the integration of these cues. Individuals with Parkinson’s disease (PD) have a particular difficulty with internally generating intentional movements and maintaining the speed, size, and vigor of movements. This difficulty improves when they are provided with external cues suggesting that there is a problem with the internal motivation of movement in PD. The prevailing view attributes this difficulty in PD to the dysfunction of motor cortical-basal ganglia circuits. First, we argue that the standard cortical-basal ganglia circuit model of motor dysfunction in PD needs to be expanded to include the insula which is a major hub within the limbic circuits. We propose a neural circuit model highlighting the interaction between the insula and dorsomedial frontal cortex which is involved in generating intentional movements. The insula processes a wide range of sensory signals arising from the body and integrates them with the emotional and motivational context. In doing so, it provides the impetus to the dorsomedial frontal cortex to initiate and sustain movement. Second, we present the results of our proof-of-concept experiment demonstrating that the functional connectivity of the insula-dorsomedial frontal cortex circuit can be enhanced with neurofeedback-guided kinesthetic motor imagery using functional magnetic resonance imaging in subjects with PD. Specifically, we found that the intensity and quality of body sensations evoked during motor imagery and the emotional and motivational context of motor imagery determined the direction (i.e., negative or positive) of the insula-dorsomedial frontal cortex functional connectivity. After 10–12 neurofeedback sessions and “off-line” practice of the successful motor imagery strategies all subjects showed a significant increase in the insula-dorsomedial frontal cortex functional connectivity. Finally, we discuss the implications of these results regarding motor function in patients with PD and propose suggestions for future studies.

## Introduction

Parkinson’s disease (PD) is a neurodegenerative disorder characterized by the degeneration of dopamine-producing neurons in the substantia nigra *pars compacta* (Braak and Braak, 2000). A major cause of morbidity in patients with PD is the difficulty sustaining a steady motor performance. This is characterized by a rapid progressive decrement in the speed, amplitude, or force of movements, and impairs everyday motor functioning of patients with PD (e.g., gait, speech, handwriting) (Chee et al., 2009; Kang et al., 2011; Ling et al., 2012). Dopaminergic treatment falls short in reversing the decrement (Espay et al., 2011). It has been shown that the decrement is most pronounced when patients with PD have to internally generate movement and improves when they are provided external cues for movement (Demirci et al., 1997; Morris et al., 2008; Tinaz et al., 2016c). For example, patients with PD can improve the progressive decline in their stride length while walking when provided with horizontal stripes on the floor (Morris et al., 2008). In a repetitive hand squeeze task using a hand clench dynamometer, we also demonstrated that patients with PD, while on dopaminergic medication, showed rapid decrement in muscle force compared with controls. This decrement was reversed when they were provided visual feedback on their performance (Tinaz et al., 2016c).

Various mechanisms have been proposed that are relevant in understanding the decrement: (1) Deficit in scaling in patients with PD is thought to contribute to the under scaling of the desired movement (Demirci et al., 1997; Maschke et al., 2003; Konczak et al., 2009). According to this view, the whole sensorimotor apparatus is “tuned-down” in PD, i.e., motor command and output as well as kinesthesia (i.e., perception of limb and body movement) are reduced. Motor performance improves when there is an external reference that allows patients with PD to make corrective adjustments. (2) Behavioral studies and computational models based on the “cost–benefit” model of motor control suggest that the energetic cost of motor performance is a major determinant of motor vigor. Higher energetic cost, if not balanced with increased motivation, would lead to scaling down of motor vigor (i.e., preference for low-effort actions). According to this framework, patients with PD assign implicitly (i.e., out of awareness) a higher energetic cost to a motor task and scale down their motor vigor (speed, amplitude, or force) (Mazzoni et al., 2007; Baraduc et al., 2013; Salimpour et al., 2015). In other words, there is a problem with internal motivation of movement in patients with PD (Mazzoni et al., 2007).

The prevailing view on the neural underpinnings of the difficulty with internally generated sustained movement in PD implicates the dysfunction of motor cortical-basal ganglia circuits. This dysfunction is attributed to the failure of basal ganglia output to reinforce the cortical mechanisms that prepare and execute the commands to move (Berardelli et al., 2001). The dorsomedial frontal cortex regions, including the supplementary motor area (SMA), pre-SMA, and cingulate motor areas, are involved in intentional motor control. Numerous neuroimaging studies have shown deficient recruitment of these regions and the basal ganglia during internally generated sequential movements in patients with PD. A common finding in these studies was the relative hyperactivation in the lateral premotor and parietal cortices which was interpreted as compensatory recruitment (Jahanshahi et al., 1995; Samuel et al., 1997; Catalan et al., 1999; Sabatini et al., 2000; Nakamura et al., 2001; Yu et al., 2007). The resting-state functional connectivity of these areas was also reduced in patients with PD compared with controls (Tinaz et al., 2016a,b). However, as we reviewed in the previous paragraph, initiating and sustaining movements require an internally driven mechanism (i.e., “cues”) for not only motor, but also motivational and sensory preparedness (Chaudhuri and Behan, 2000). In addition to the motor cortical-basal ganglia circuits, the limbic circuits also play a role in the integration of these cues. Therefore, it is conceivable that the disrupted integration in both motor and limbic circuits in PD may lead to defective cue production for initiating and sustaining movements. Yet, the potential role of dysfunction in limbic circuits pertaining to the internal drive behind intentional movement has been under-investigated in PD.

**FIGURE 1:**
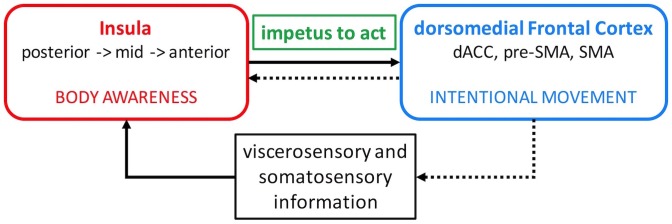
Insula – dorsomedial frontal cortex interaction model. Viscerosensory and somatosensory afferent information is relayed to the insula where it is processed along a posterior-to-anterior axis and integrated with emotional salience and motivational potential. This elaborate information about the body is conveyed to the dorsomedial frontal cortex and generates the impetus to move (solid arrow). The insula then evaluates the outcome of the intentional movement to reinforce adaptive movements in the future (dotted arrows). dACC, dorsal anterior cingulate cortex; SMA, supplementary motor area.

In the first part of this paper, we argue that the standard cortical-basal ganglia circuit model of motor dysfunction in PD needs to be expanded to include the insula which is a major hub within the limbic circuits. The insula is involved in processing a wide range of sensory signals arising from the body and integrates them with the emotional and motivational context. In doing so, it is thought to provide the impetus for intentional movement. We propose a neural circuit model highlighting the interaction between the insula and dorsomedial frontal cortex in this process (Figure 1). In the second part, we present the results of our proof-of-concept experiment demonstrating that this interaction can be enhanced non-invasively with neurofeedback-guided motor imagery using functional magnetic resonance imaging (fMRI) in patients with PD. Finally, we discuss the implications of these results regarding motor function in patients with PD and propose suggestions for future studies.

### Insula Is Important for Sustained Intentional Movement

Intentional movement is an internally driven process. In addition to the motor cortical-striatal regions, the insula is also thought to play a role in this process. The insula is a multifaceted limbic cortex with rich anatomical interconnectivity and functional diversity. Distinct subdivisions of the insula process information ranging from visceral and somatic sensations and emotional states to motor and cognitive control (Nieuwenhuys, 2012; Uddin et al., 2014; Nomi et al., 2016). Specifically, the insula plays a fundamental role in body awareness. Here, we use body awareness as an umbrella term which encompasses interoceptive (i.e., subjective sense of the physiological condition of the entire body) and somatosensory (e.g., touch, kinesthesia, proprioception) awareness (Craig, 2002, 2003, 2009; Critchley et al., 2004; Kurth et al., 2010; Mehling et al., 2012; Garfinkel et al., 2015; Kenzie et al., 2016). Viscerosensory afferents carrying interoceptive information (e.g., hunger, thirst, heartbeat, bowel or bladder distention, body temperature, pain, itch, muscle ache) via the vagal and glossopharyngeal cranial nerves and the spinothalamic tract converge in the nucleus tractus solitarius in the brainstem. These visceral inputs are then relayed to the ventromedial posterior nucleus of the thalamus, and from there projected onto the amygdala, insula, and the cingulate cortex (Critchley, 2005; Critchley and Harrison, 2013; Critchley et al., 2013). Somatosensory information from the thalamus and somatosensory cortex is also processed in a similar fashion within the insula (Dijkerman and de Haan, 2007; Kurth et al., 2010). The interoceptive and somatosensory information is thought to be transmitted following a gradient along the caudal to rostral axis of the insula. This information is first processed in the posterior insula and relayed to the mid and anterior insula where it is integrated with emotional salience and motivational potential, which are represented in brain regions to which the mid and anterior insula are connected (Craig, 2002, 2009; Strigo and Craig, 2016). Another important component of body awareness is the sense of body ownership (i.e., the sensation that different body parts belong to one’s self). Sense of body ownership is present during externally generated bodily experiences (e.g., experiencing the other’s pain in one’s own body) (Bucchioni et al., 2016) as well as during voluntary actions which create a sense of self-agency (Tsakiris and Haggard, 2005). The insula plays a prominent role in sense of body ownership and of self-agency (Karnath and Baier, 2010).

The dorsal anterior and mid insula have strong connections with the mid-cingulate cortex, pre-SMA, and SMA (Ghaziri et al., 2017) which are dorsomedial frontal areas involved in intentional motor control including decisions regarding which action to perform and when to perform it (Picard and Strick, 2001; Hoffstaedter et al., 2014; Zapparoli et al., 2017). Activation in these insula regions also correlates with the sense of self-agency during voluntary motor tasks (Farrer and Frith, 2002). Moreover, intentional decision on whether or not to act activates the right insula and right anterior and left mid cingulate cortex (35). It has been proposed that the elaborate and contextualized information about the body processed in the insula is relayed to the dorsomedial frontal cortex and used to initiate new or modify ongoing actions (Paulus et al., 2009; Brass and Haggard, 2010). In this manner, body awareness can generate the impetus to act. The insula then evaluates the outcome of the intentional action to reinforce adaptive actions in the future (Brass and Haggard, 2010). Therefore, it seems that an appropriate level of insula interaction with the dorsomedial frontal cortex is necessary to initiate and continue action (Figure 1).

In addition to the dorsomedial frontal cortex regions, the insula is also impacted by the pathological process in PD structurally and functionally. According to the Braak classification, the insula is one of the first cortical regions affected by alpha-synuclein aggregates (Braak et al., 2006). Moreover, using fMRI and graph theory-based network analysis, we and others demonstrated significantly reduced resting-state functional connectivity in the insula in patients with PD compared with controls (Koshimori et al., 2016; Tinaz et al., 2016a). The betweenness centrality, a network measure of how well a region behaves as a hub, of the insula was also significantly reduced in patients with PD (Koshimori et al., 2016; Tinaz et al., 2016a). This abnormal connectivity correlated with the severity of symptoms and motor signs (Tinaz et al., 2016a).

In summary, we propose that the interaction between the insula and dorsomedial frontal cortex plays an important role in internally generating and sustaining intentional movements. These regions are major hubs in the limbic and motor networks, respectively, and are impacted by the pathological process in PD.

### Hypothesis Testing: The Activity of the Insula-Dorsomedial Frontal Cortex Circuit Can Be Enhanced With Neurofeedback Training in Subjects With PD

We hypothesize that fMRI-based neurofeedback can be used as a non-invasive intervention to enhance the functional connectivity between the insula and dorsomedial frontal cortex in subjects with PD. Neurofeedback enables subjects to obtain voluntary control over their brain activity. With practice, subjects also learn to regulate the behavior that is associated with this brain activity. FMRI-based neurofeedback has been used successfully in symptom treatment in several neuropsychiatric disorders (e.g., anxiety, depression, and addiction) (Linden et al., 2012; Li et al., 2013; Scheinost et al., 2013a) and in PD (Subramanian et al., 2016).

In this study, we employed a technique that used the functional connectivity strength between the right insula and dorsomedial frontal cortex, as opposed to the activity in either region alone, as neurofeedback (Megumi et al., 2015; Koush et al., 2017). We chose motor imagery as the mental strategy for neurofeedback learning in subjects with PD. This choice warrants further explanation:

Motor imagery refers to the mental rehearsal of motor acts without overt body movement and recruits virtually the same brain regions that are involved in the actual planning and execution of motor tasks (Guillot et al., 2014). The duration of the imagined movements correlates with that of the real movements. Imagined and real movements also evoke similar autonomic responses. These similarities led to the notion of functional equivalence which likely explains the beneficial effect of motor imagery on motor performance in athletes (Guillot and Collet, 2008) and in rehabilitation of neurological disorders (e.g., stroke) (Di Rienzo et al., 2014). Surprisingly, motor imagery practice has been rarely employed in the motor rehabilitation of patients with PD, partly due to the discouraging viewpoint about its utility in PD (Dickstein and Tamir, 2010). However, one study demonstrated significant improvement in slowness during sequential movement tasks in patients with PD who received 12 weeks of motor imagery practice of everyday actions compared with the control group (Tamir et al., 2007). Neuroimaging studies in PD demonstrated reduced activation in the dorsomedial frontal cortex regions during motor imagery which was improved with dopaminergic treatment (Dickstein and Tamir, 2010). In addition, a randomized trial using fMRI-based neurofeedback with motor imagery in patients with PD (*N* = 15) demonstrated significant increase in the SMA activity (Subramanian et al., 2016). These findings suggest that patients with PD have the capacity to use motor imagery in neurofeedback learning. They can also benefit from its practice when the imagery tasks focus on activities of daily life to re-activate motor representations that are part of the patient’s motor repertoire (Dickstein and Tamir, 2010).

Importantly, motor imagery content determines the brain activation patterns. Kinesthetic motor imagery (i.e., mental image of the sensation of movement) evokes sensorimotor simulations of one’s own body and preferentially recruits the sensorimotor-related brain regions including the insula (Lorey et al., 2009), whereas visual motor imagery (i.e., seeing the movement in mind’s eye) preferentially recruits the visuospatial-related brain regions (Guillot et al., 2009, 2014). Kinesthetic motor imagery has been used successfully in healthy subjects during neurofeedback learning to enhance the activation in sensorimotor brain regions (Marchesotti et al., 2016). Kinesthetic motor imagery also fits our insula (body awareness) – dorsomedial frontal cortex (movement) interaction model (Figure 1). We hypothesize that subjects with PD can use this strategy successfully during neurofeedback training to increase the functional connectivity strength between the right insula and dorsomedial frontal cortex.

In summary, in this proof-of-concept study we used fMRI-based neurofeedback training in a group of subjects with mild PD while they performed kinesthetic motor imagery, and tested their ability to learn to increase the right insula-dorsomedial frontal cortex functional connectivity. In the next section, we summarize the methods and results of our study.

## Materials and Methods

**FIGURE 2:**
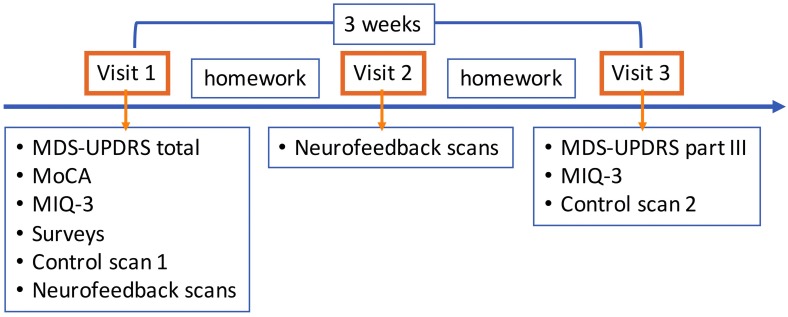
Neurofeedback study flow. Visit 1 consisted of clinical assessments and self-evaluation surveys, motor imagery strategy development session, first control scan without neurofeedback, and neurofeedback scans (4–5). Successful motor imagery strategies during neurofeedback training were assigned as motor imagery homework, and subjects were provided diaries to keep a log. Only neurofeedback scans (6–7) were performed in visit 2. Subjects were again assigned motor imagery homework. In visit 3, MDS-UPDRS part III and MIQ-3 were repeated and the second control scan without neurofeedback was performed. The time between the first and last visits was on average 3 weeks. MDS-UPDRS, Movement Disorders Society Unified Parkinson’s Disease Rating Scale; III, Part III motor exam; MIQ-3, Movement Imagery Questionnaire-3.

All subjects participated in the study after giving written informed consent in accordance with the procedures approved by the Human Research Protection Office of the Yale School of Medicine. Subjects were recruited primarily through the Connecticut Advocates for Parkinson’s group and the Movement Disorders Clinic at the Yale School of Medicine. The study was conducted at the Yale Magnetic Resonance Research Center. All subjects underwent an initial screening for MRI safety and medical history. There were two independent groups of subjects with PD. The first group (*N* = 10) participated in a heartbeat counting task adapted for use in fMRI. The second group (*N* = 8) participated in the neurofeedback study within a 3-months enrollment period (Figure 2).

### Subjects

Subjects with a diagnosis of idiopathic PD according to the United Kingdom Parkinson’s Disease Society Brain Bank Clinical Diagnosis Criteria (Hughes et al., 1992) and on a stable dopaminergic medication regimen were recruited. Subjects with PD who were not fully independent; had a neurological or psychiatric disorder (other than PD and comorbid depression or anxiety), or a medical condition that might affect the central nervous system, history of alcohol or illicit drug abuse, head injury resulting in loss of consciousness, dementia (Montreal Cognitive Assessment score < 21), or contraindications for MRI were excluded.

Disease severity was assessed using the Movement Disorders Society Unified Parkinson’s Disease Rating Scale (MDS-UPDRS) (Goetz et al., 2008) and the Hoehn and Yahr (H & Y) scale (Hoehn and Yahr, 1967). The cut-off for H & Y for inclusion was ≤2.5 (i.e., mild bilateral disease with some impairment in balance). Subjects in the heartbeat counting group were scanned in the morning when they were off of dopaminergic medications for 12 h (practical “off” state). Subjects in the neurofeedback group were scanned in the morning after their first dose of dopaminergic medication. Neurological and neuropsychological assessments were performed by a neurologist (S.T. or A.V.R.) prior to scanning.

### Self-Evaluation Questionnaires

Emotional and motivational state and fatigue levels can influence the motor imagery performance. The levels of anxiety, depression, apathy, fatigue, and overall quality of life were assessed using standardized questionnaires on visit day 1 (see Supplementary Material for details). Motor imagery skills were assessed using the Movement Imagery Questionnaire-3 (MIQ-3) (Williams et al., 2012). The MIQ-3 is an examiner-administered questionnaire that requires subjects to perform four complex movements, then imagine the movements and rate the difficulty of motor imagery on a Likert-type scale from 1 (very hard) to 7 (very easy).

### Scanning

#### MRI Sequences

Scanning was performed in a 3.0 Tesla Siemens Trio TIM human research MRI scanner using a 32-channel head coil.

High-resolution T1-weighted MPRAGE anatomical images (176 slices, slice thickness: 1 mm, in-plane resolution: 1 mm × 1 mm, FoV: 250 mm, Matrix: 256 × 256, TR: 1900 ms, TE: 2.52 ms, TI: 900 ms, flip angle: 9 degrees) were collected for an accurate localization of the fMRI data in the beginning of each scan session. T1-weighted FLASH axial images (36 slices, slice thickness: 4 mm, no spacing; in-plane resolution: 0.9 mm × 0.9 mm, FoV: 224 mm, Matrix: 256 × 256, TR: 300 ms, TE: 2.47 ms, flip angle: 60 degrees) were collected as an intermediate scan to coregister MPRAGE and echo planar functional images for the neurofeedback sessions. Then, axial T2-weighted, echo planar functional images were collected (36 slices, slice thickness: 4 mm, no spacing; in-plane resolution: 3.5 mm × 3.5 mm, FoV: 224 mm, Matrix: 64 × 64, TR: 2000 ms, TE: 25 ms, flip angle: 90 degrees). A short functional localizer scan (10 s) with the same parameters was also collected for registration purposes only for the neurofeedback sessions.

The number of functional volumes was 134 (4 min 28 s) for the heartbeat counting task and 120 (4 min) for the neurofeedback task.

#### Heartbeat Counting Task

Subjects in the first PD group performed a silent heartbeat counting task which they practiced first outside the scanner. This task is commonly used to assess interoceptive awareness and has been shown to activate particularly the right insula and dorsomedial frontal cortex in healthy subjects (Critchley et al., 2004; Pollatos et al., 2007; Kurth et al., 2010; Simmons et al., 2013; Schulz, 2016). It was used here to functionally localize the right insula and dorsomedial frontal cortex and create anatomical masks for use in the neurofeedback experiment.

During the heartbeat counting block (30 s), subjects focused on their heartbeat and tried to count it silently without checking their pulse. In the end of the counting block, they chose a respond button to indicate their count (6 s). There were four blocks in each run and three runs in total (see Supplementary Material).

#### Neurofeedback Task

##### Strategy development session

The purpose of this session was to determine each subject’s motor repertoire, identify their motor difficulties, and familiarize them with motor imagery practice. Personalized motor imagery strategies were discussed to provide a context for the subjects. Subjects were instructed to use motor imagery of whole body complex movements that are part of their motor repertoire while at the same time focusing on the imagined bodily felt sense (e.g., proprioceptive, kinesthetic, autonomic, etc.) that these movements evoke. Subjects were also primed to experience body awareness by engaging in a mindfulness body scan practice for 11 min during which they listened to an audio recording guiding them to pay attention to sensations in different body parts.

##### Neurofeedback paradigm

**FIGURE 3:**
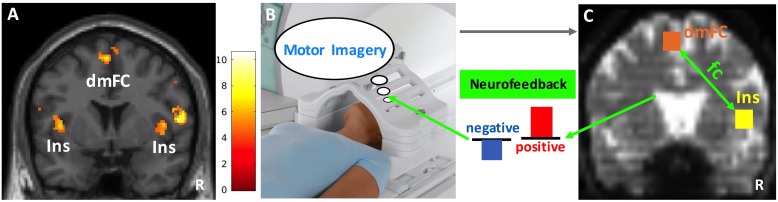
Neurofeedback paradigm. **(A)** The insula (Ins) and dorsomedial frontal cortex (dmFC) group activations (*N* = 10) during the heartbeat counting task are shown on a coronal slice of the canonical MNI brain template. Color bar represents the *t*-values. **(B)** Functional scans during motor imagery (40 s) were collected and preprocessed in real-time. **(C)** Cubic anatomical masks (6 mm × 6 mm × 6 mm) were centered at the voxel peak activity in the right insula and dorsomedial frontal cortex. These masks were created in the standard MNI space and then translated into each subject’s functional space. The signal time courses averaged across voxels within each mask were correlated with each other to compute the functional connectivity (fc) between right insula and dorsomedial frontal cortex. Finally, the z-transformed correlation values were plotted as bars to provide the neurofeedback (blue: negative, red: positive). The size of the bars reflects the magnitude of neurofeedback. R, right. Image in **(B)** depicts a Siemens Tx/Rx CP head coil (www.healthcare.siemens.com).

A night sky picture on the screen instructed subjects to engage in motor imagery for 40 s. This picture with subdued visual input was chosen to minimize interference during imagery. Subjects were told not to change strategies within a block. The right insula-dorsomedial frontal cortex functional connectivity strength was computed during the 40-s task block and presented to the subject in the form of a bar plot for 8 s at the end of the block to provide neurofeedback (blue bar: negative, red bar: positive neurofeedback) (Figures 3B,C). The magnitude of the bar reflected the strength of the functional connectivity, and subjects were instructed to increase this. There were five motor imagery task blocks followed by feedback in each session. Each subject completed a total of 10–12 sessions on visit days 1 and 2. We used intermittent neurofeedback at the end of each task block (Johnson et al., 2012) because his method has the following advantages over continuous neurofeedback: (1) Subjects do not need to be aware of the 6–8 s hemodynamic delay, (2) the potentially distracting confound of cognitive load associated with constant feedback monitoring can be avoided, and (3) brain activity related to feedback evaluation and actual task performance can be separated out.

The control scans without neurofeedback on the first and last visit days were implemented in the same way, but a horizontal white line was presented for 8 s after each task block. Subjects were aware that they were not going to receive feedback on their performance during control scans. The difference in performance between the first and last control scan served as a measure of learning.

### Motor Imagery Homework

After the neurofeedback scans on visit day 1, a detailed first-person account of each subject’s experience during neurofeedback training and a list of strategies were documented. Subjects were told to practice the motor imagery strategies that generated positive feedback for about 10–15 min everyday until their next visit. Subjects were also assigned to engage in the mindfulness body scan exercise daily to prime their motor imagery practice. They were provided with a diary to keep a log of their motor imagery practice reporting on whether they practiced the mindfulness body scan, duration of motor imagery practice, motor imagery environment and content, the reason why specific content was chosen, the associated body sensations and their quality during motor imagery, and the difficulty level of motor imagery. This was an iterative process. The diary entries were reviewed to further refine the strategies for use during neurofeedback learning on visit day 2, and subjects were again instructed to practice the successful motor imagery strategies at home until the last visit.

### Data Analysis

#### Behavioral Data Analysis

##### Questionnaires

The distribution of scores was first tested for normality using the Shapiro–Wilk statistics. Mean (for normally distributed) or median (for not normally distributed) scores were compared to the normative scores using a one-sample *t*-test (for normally distributed) or Wilcoxon *t*-test (for not normally distributed) in SPSS 24 (see Supplementary Material).

The MDS-UPDRS part III motor exam and MIQ-3 scores before and after neurofeedback training were compared using a paired-sample *t*-test (*p* < 0.05, two-tailed).

##### Motor imagery diaries

Subjects were given multiple choices from which they could select to describe their motor imagery experience and were also free to add their own descriptions. The entries were coded and organized under categories. This approach was similar to a data-driven and descriptive thematic analysis (Braun and Clarke, 2006). The purpose was to catalog the first-person accounts of the whole group.

#### Imaging Data Analysis

##### Heartbeat counting task

SPM12 was used for analysis (Penny et al., 2007). Preprocessing steps included the removal of the first four scans to reach magnetization steady state, motion correction, coregistration of functional scans with the anatomical scan, normalization to the standard MNI template, and smoothing of the functional scans with a 6-mm kernel. Cut-off for translational motion amount was less than one voxel for all subjects. The general linear model was used for the for the first-level analysis. The task blocks were convolved with the canonical hemodynamic response function. The model included grand mean scaling, high-pass filtering at the cut-off frequency of 1/128 Hz, the AR1 method of estimating temporal autocorrelation, and time derivatives. The main contrast of interest was the heartbeat counting > baseline activity. A second-level group analysis was performed using a one-sample *t*-test on the heartbeat counting blocks (*p* < 0.001, uncorrected, cluster size = 10). Small volume correction was also applied to the right insula and dorsomedial frontal cortex activation peaks using a sphere with a radius of 15 mm.

##### Neurofeedback task

We created cubic anatomical masks (6 mm × 6 mm × 6 mm) centered at the voxels that showed peak activity in the right insula and dorsomedial frontal cortex during the heartbeat counting task for use in neurofeedback training (see section “Heartbeat Counting Task,” right insula peak: *x* = 44, *y* = 4, *z* = 8 and dorsomedial frontal cortex peak: *x* = -4, *y* = 2, *z* = 62). These masks were created based on the standard MNI brain template and then translated into each subject’s native functional space using a series of transformations (Figure 3C). All transformations were estimated using Bioimage Suite and manually inspected for accuracy. Functional scans of each subject were motion corrected in real-time using the algorithms described in Scheinost et al. (2013b). Covariates of no interest were regressed out including linear and quadratic drifts; mean cerebral spinal fluid, white matter, and gray matter signals; and motion-related confounds (24-parameter motion model including six rigid-body motion parameters, six temporal derivatives, and these terms squared). The data were temporally smoothed with a zero-mean unit variance Gaussian filter (approximate cutoff frequency = 0.12 Hz). Then, the signal time course of the right insula and dorsomedial frontal cortex masks in a given subject were computed as the average time course across all voxels within each of these masks. Finally, the time courses were correlated and the *r*-values were Fisher *z*-transformed. A Matlab program plotted the *z*-values as a bar graph and presented them as neurofeedback.

The significance of the difference in functional connectivity between the second and first control scans was evaluated using a paired-sample *t*-test (*p* < 0.05, two-tailed).

## Results

### Demographic and Clinical Data

Demographic data and the mean scores and standard deviations of the clinical data for the neurofeedback PD group are summarized in Table 1 (see also Supplementary Table S1 for the first PD group). The neurofeedback PD group did not have significant anxiety, depression, fatigue, or apathy. The quality of life scores were significantly lower (i.e., better quality of life) than the normative mean established for patients with PD. A detailed description of the results including normative data and cut-off scores can be found in the Supplementary Material.

**Table 1:** Demographic and clinical data.

Gender	4 Male; 4 Female
Age	66.0 ± 8.5
Disease onset side	5 right, 3 left
Disease duration (years)	3.0 ± 2.5
LEDD (mg)	364.1 ± 292.0
MDS-UPDRS total	44.8 ± 5.4
MDS-UPDRS III	32.1 ± 6.6
H&Y	2.0 ± 0
MoCA	26.5 ± 1.9
STAI-T	35.9 ± 12.9
STAI-S	27.8 ± 4.7
BDI-II	7.6 ± 6.3
Apathy	11.3 ± 5.4
PFS	37.1 ± 12.8
PDSI	13.8 ± 12.3


*LEDD, levodopa equivalent dose; MDS-UPDRS*, *Movement Disorders Society Unified Parkinson’s Disease Rating Scale; III, Motor exam scores; H & Y, Hoehn & Yahr; MoCA, Montreal Cognitive Assessment battery; STAI-S and STAI-T, Spielberger State-Trait Anxiety Inventory; BDI-II, Beck Depression Inventory-II; PFS, Parkinson’s Fatigue Scale; PDSI, Parkinson’s Disease Summary Index calculated based on the PDQ-39 scores (see Supplementary Material)*.

On average, subjects found motor imagery “somewhat easy”-to-“easy” to perform. The difference in motor imagery difficulty ratings before and after neurofeedback training was not significant (see Supplementary Material).

The mean MDS-UPDRS part III motor exam score was 32.1 ± 6.6 at baseline and 31.8 ± 4.5 after neurofeedback training. This difference was not statistically significant (*p* = 0.871).

### Imaging Results

#### Heartbeat Counting Task

Group analysis of ten subjects revealed activation in the right insula (peak coordinates: *x* = 44, *y* = 4, *z* = 8, *Z* = 3.91; small volume corrected p_FWE_ = 0.046) and dorsomedial frontal cortex (peak coordinates: *x* = -4, *y* = 2, *z* = 62, *Z* = 4.39; small volume corrected p_FWE_ = 0.010) (Figure 3A). There was also significant activation in the left insula and motor/premotor, occipital, and parietal areas (see Supplementary Figures S1, S2 and Supplementary Tables S2, S3 for details).

#### Neurofeedback

**FIGURE 4:**
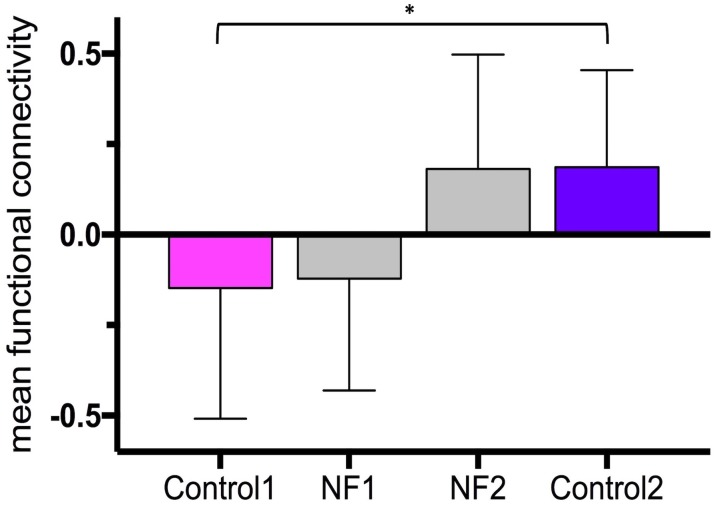
Neurofeedback learning. The bar graphs show the mean right insula-dorsomedial frontal cortex functional connectivity (i.e., z-transformed correlation values) averaged across subjects and task blocks. Control 1, first control scan without neurofeedback; Control 2, last control scan without neurofeedback; NF 1, neurofeedback scans on day 1; NF 2, neurofeedback scans on day 2. ^∗^*p* = 0.009.

The average time between the first and last control scans was 21.5 ± 2.6 days. The average time between the last dose of medication and start of the neurofeedback scans was 2 h 26 min ± 1 h 6 min. The bar graphs in Figure 4 show the mean and standard deviation of *z*-values of the right insula-dorsomedial frontal cortex functional connectivity averaged across task blocks and subjects (mean *z*-values of the first control scan: -0.15 ± 0.36, of neurofeedback scans on day 1: -0.12 ± 0.31, of neurofeedback scans on day 2: 0.18 ± 0.32, and of the second control scan: 0.19 ± 0.27). The mean *z*-values across subjects were normally distributed. The paired-sample *t*-test revealed a significant increase in the mean *z*-values from the first to the second control session (*p* = 0.009).

### Motor Imagery Subjective Reports

#### During Neurofeedback Learning

We found that the strategies commonly used by all subjects were motor imagery of coordinated whole body movements including everyday activities and exercise routines: walking (e.g., around the neighborhood, on the beach, hike), running (e.g., marathon, on the beach, treadmill), combinations of calisthenics (e.g., jumping jacks, jumping rope, sit-up, push-up, squat, lunge), lifting weights, boxing, “big” movements of “Big and Loud” exercises, swimming (e.g., breaststroke, crawl), shoveling snow, raking leaves, cutting wood.

Importantly, based on subjective reports, two additional specific factors emerged that determined the direction of the neurofeedback. In other words, motor imagery of the same complex movements could generate positive or negative neurofeedback (i.e., positive or negative functional connectivity between the right insula and dorsomedial frontal cortex) depending on these two factors:

(1) The quality and vividness of body sensations during motor imagery:
  For example, feeling the movement, stretch, and weight in joints and muscles, feeling the impact of a punch or kick, feeling the rhythm of movement, awareness of the heartbeat and breathing, and awareness of externally triggered sensations (e.g., feeling the sand or water on the body or the warmth of sunshine) were associated with positive neurofeedback. On the other hand, motor imagery that was mostly visual (e.g., seeing self in action from outside) or did not elicit much kinesthetic awareness (e.g., doing the movement, but not feeling it) and awareness of physical limitations and body discomfort (e.g., awareness of muscle stiffness or slowness during imagery of walking, swimming, etc.) were associated with negative neurofeedback.
(2) The emotional and motivational valence of motor imagery:
  Motor imagery that was performed with a sense of ease, relaxation, pleasure, enjoyment, and accomplishment was associated with positive neurofeedback, whereas motor imagery that was performed with lack of enjoyment or confidence, or a sense of frustration and failure was associated with negative neurofeedback.

In addition, mind wandering, distraction, mental fatigue, and actual disruptive somatic sensations (e.g., tremor, muscle stiffness, discomfort from lying flat in the scanner) during motor imagery were associated with negative neurofeedback.

#### Diaries

On average, there were 14.1 ± 4.4 motor imagery entries. The mean duration of each motor imagery practice was 13.6 ± 7.5 min. The mean difficulty level of motor imagery performance was 3.4 ± 0.3 (5: very easy, 4: easy, 3: neutral, 2: difficult, 1: very difficult). Difficulty staying focused rather than motor imagery itself was the main challenge reported by the subjects.

Subjects reported that most of the time they also listened to the mindfulness body scan audio prior to motor imagery. The motor imagery contents and body sensations in individual diaries were very similar to those compiled after neurofeedback training. Table 2 summarizes the diary entries (*n* = 111) and the frequency (%) of reporting. A particular activity or movement during imagery was chosen mainly for the following reasons: (1) Needs improvement (e.g., balance, strength, coordination, stamina, performance of a particular movement or exercise), (2) Positive emotions and attitude associated with the imagined activity (e.g., fun, good, great, enjoyable, pleasant, rewarding, relaxing, emotionally satisfying, peaceful, sense of accomplishment), and (3) Familiarity with the imagined movements or activity (e.g., daily exercise, part of daily routine).

**Table 2:** Summary of motor imagery diary entries.

Imagined movements/activities	%	Reason of motor imagery choice	%
Calisthenics	35	Need to improve	49
Walking	29	Positive emotions and attitudes	38
“Big” exercises	14	Familiarity	18
Weight lifting	12		
Balance/coordination	11		
Everyday activities (e.g., shoveling snow)	8		
Other (e.g., boxing, yoga, and swimming)	14		
**Body sensations evoked during imagery**	**%**	**Quality of body sensations**	**%**
Kinesthesia	86	Pleasant	72
Breathing	80	Comfortable	57
Heartbeat	53	Soothing	23
Touch/pressure/stretching	26	Other positive (e.g., refreshing)	5
Stiffness	13	Uncomfortable	37
Tremor	9	Distracting	15
Pain	8	Other negative (e.g., tiring and challenging)	7
Other (e.g., rhythm and temperature)	14		


Subjects also recorded descriptions of their overall experiences:

“I could do jumping jacks with more control in class today, imagery seems to help.”

“Splitting large oak logs: I expected the task to be nearly impossible in reality; [after motor imagery], it was doable in reality.”

“If I do activities immediately following imagery, I have greater success; body scan helps me to relax and focus thoughts; best to do it when I don’t have a lot on my plate; similar to meditation- when performing activities that are difficult due to Parkinson’s, I can sometimes create body scan environment in my mind, resulting in improved performance.”

## Discussion

In summary, our group included subjects with mild bilateral PD who, as a group, did not have significant apathy, anxiety, depression, or fatigue. All subjects learned to increase the right insula-dorsomedial frontal cortex functional connectivity using neurofeedback-guided motor imagery. Next, we elaborate on the results of our proof-of-concept neurofeedback experiment in relation to our hypothesis and neural circuit model, discuss the therapeutic implications of these results, and offer suggestions for future studies.

### Functional Localizer Task

Our group results based on ten PD subjects are in line with previous reports demonstrating bilateral mid insula (right > left) and dorsomedial frontal cortex activation during heartbeat counting tasks (Critchley et al., 2004; Pollatos et al., 2007; Kurth et al., 2010; Simmons et al., 2013; Schulz, 2016). There was heterogeneity in the location of the peak insula activation across subjects including the anterior and mid insula, and activation often extended to the adjacent frontal operculum. Similar heterogeneity has been reported in previous studies. One factor that might be contributing to this heterogeneity is that imaging studies to date have used somewhat different interoception tasks that rely on different processes. For example, the silent heartbeat counting task depends on internal monitoring mechanisms whereas the heartbeat discrimination task requires integration of internal and external cues (Garfinkel et al., 2015). In the heartbeat discrimination task, subjects have to simultaneously keep track of their heartbeat and the accuracy of the external stimulus yoked to their pulse. Attention to the external stimulus may recruit the anterior insula and dorsal anterior cingulate cortex more strongly (Critchley et al., 2004) both of which are the major hubs of salience detection (Menon and Uddin, 2010). We used the silent heartbeat counting task without any external reference to avoid attentional overload in PD subjects and did not observe anterior cingulate cortex, but SMA activation extending to the pre-SMA. The coordinates of the SMA/pre-SMA activation in our study strongly overlap with those reported in previous studies of interoception (Critchley et al., 2004; Kurth et al., 2010; Simmons et al., 2013; Schulz, 2016) further supporting the view that this region typically involved in intentional motor control (Picard and Strick, 2001) is also recruited during monitoring of the internal milieu (Paulus et al., 2009; Brass and Haggard, 2010).

Finally, the right insula and dorsomedial frontal cortex group activation peaks survived small volume correction suggesting that the functional localization of these peaks was reasonably reliable despite inter-subject variability.

### Specificity of Learning With Neurofeedback-Guided Kinesthetic Motor Imagery

All subjects showed a learning effect. We did not include a sham (i.e., non-contingent) neurofeedback condition as a control, therefore, one may question whether learning was specific to neurofeedback or related to some other general factor such as motivation or attention. However, several key elements and observations in our study point to a contingent learning effect in a precise context: (1) We provided explicit instructions for a specific mental strategy for use during neurofeedback training (i.e., engage in kinesthetic motor imagery and focus on body sensations). Subjects were given freedom to choose their individual scenarios within this context. In other words, self-regulation during neurofeedback learning was constrained to this context. (2) Detailed subjective reports also suggest mapping of the mental strategies onto the probed neural circuit during neurofeedback learning. Of note, based on subjective reports, kinesthetic motor imagery with vivid awareness of positive body sensations elicited positive neurofeedback, whereas motor imagery that was mostly visual, did not elicit much body awareness, or was associated with awareness of negative body sensations generated negative feedback. The emotional and motivational valence of motor imagery (e.g., frustration versus pleasure) was also important in determining the direction of neurofeedback. These results corroborate our neural circuit model and demonstrate the specificity of neuromodulation. Namely, the insula processes the viscerosensory and somatosensory signals arising from the body, thereby creating body awareness, and integrates them with the emotional and motivational context. The valence of this context, in which body awareness is embedded, plays an important role in insula’s relationship with the dorsomedial frontal cortex. A positively valenced context promotes correlated activity between the two regions, whereas a negatively valenced one does the opposite. These results support the central notion in motor imagery literature that to be efficient, motor imagery should be based on positive mental images (Guillot et al., 2014).

Furthermore, the neuroimaging findings and subjective motor imagery reports also relate to the personal experience of patients with PD during actual physical activity. Their motor difficulties (e.g., tremor, freezing of gait, festination) are exacerbated when they are anxious or emotionally distressed (Giladi and Hausdorff, 2006; Dissanayaka et al., 2010; Starkstein et al., 2015).

### Therapeutic Potential of Neurofeedback-Guided Kinesthetic Motor Imagery in PD

The subjective reports during neurofeedback learning informed and shaped the subsequent motor imagery practice at home. On average, subjects practiced motor imagery everyday for 2 weeks, each practice lasting about 13 min. Subjects gave rich accounts of their motor imagery experience including detailed descriptions of imagined movements and movement sequences as well as the body sensations. They rated motor imagery as neutral-to-easy to practice. Main challenge during practice was reported as the difficulty to maintain attention rather than motor imagery itself. The body sensations evoked during motor imagery were mostly pleasant, comfortable, and soothing. All subjects reported that listening to the mindfulness body scan audio before motor imagery helped them relax and get in touch with the body sensations. Almost half the time, subjects chose a particular motor imagery content because they wanted to improve the actual motor performance. About one third of the time, the reason was positively valenced emotions and attitude associated with the motor imagery content. These rich first-person accounts suggest that individuals with PD are capable of practicing motor imagery efficiently. More importantly, they are motivated to incorporate motor imagery into their physical activities with the goal to improve motor function.

### Limitations and Future Directions

Our proof-of-concept study has inherent limitations including the small sample size and lack of a control condition. The primary aim of the experiments was to demonstrate the testability of our hypothesis and proposed neural circuit model. Therefore, our results should be interpreted only as preliminary evidence supporting our hypothesis and need to be validated in future studies with an appropriately powered sample size and control condition. Moreover, we suggest that future studies take the following points into consideration:

Based on our finding that frustration consistently generates negative neurofeedback, we think that sham neurofeedback may not be the optimal control condition for subjects with PD. The potential risk of frustration associated with sham neurofeedback might result in an overestimation of the effects of contingent neurofeedback. An active control condition (e.g., guided mental imagery without a movement component) instead of sham neurofeedback might be more suitable to minimize this risk.

We did not find a significant difference in the MDS-UPDRS motor exam scores before and after training. In addition to the small sample size, a potential factor might be the ceiling effect. The motor exams were performed in “on-medication” state for all subjects who had mild disease and were relatively high-functioning. Off-medication comparison of the MDS-UPDRS motor exam scores might offer a more realistic assessment of potential benefit from the neurofeedback intervention. We also suggest including motor function tests to assess performance of movement sequences in clinical outcome measures (Tamir et al., 2007).

Other factors might be the duration of and compliance with off-line practice. In one study using motor imagery alone in subjects with PD, significant improvement in motor exam scores was detected after 12 weeks of training (Tamir et al., 2007). In a neurofeedback-guided motor imagery study, this improvement was achieved after a total of 11 neurofeedback training runs and off-line practice spread over 10 weeks (Subramanian et al., 2016). The optimal duration of motor imagery off-line practice needs to be determined in future studies. Compliance with off-line practice also needs to be monitored closely. Furthermore, the duration of practice should not be more than 5 min without a break (Tamir et al., 2007) to prevent mental fatigue. Providing a personalized motor imagery script might also help subjects sustain attention.

We used the MIQ-3 to evaluate the motor imagery skills. This is a subjective rating scale. In addition, the mental chronometry method to measure the duration of motor imagery would provide an objective assessment of motor imagery performance (Guillot and Collet, 2005).

It is also important to note that body awareness includes plastic components such as cognitive appraisal and emotion regulation. Cultivating these components (e.g., using contemplative and mindfulness practices) in combination with neurofeedback-guided kinesthetic motor imagery would improve adaptive emotional and motor skills in PD (Farb et al., 2015; Mehling, 2016).

## Conclusion

The insula-dorsomedial frontal cortex circuit is important in internally generating intentional movements. Neurofeedback-guided kinesthetic motor imagery increases the activity of this circuit and has the potential to be used as a therapeutic intervention to improve sustained motor performance in patients with PD. Most neuropsychiatric conditions including PD are associated with dysfunction of neural circuits rather than of individual brain areas. Therefore, using the functional connectivity between two or more brain regions, as opposed to activation in a brain region alone, as neurofeedback might be a neurobiologically more specific approach. Interventions such as deep brain stimulation surgery exert their effect by altering the abnormal activity of targeted brain circuits underlying PD pathology. Functional connectivity-based neurofeedback intervention offers an opportunity to have a similar effect on motor function non-invasively. If successful, it has the potential to be used as a personalized intervention for sustained motor benefit in patients with PD.

## Ethics Statement

This study was carried out in accordance with the recommendations of ‘Human Research Protection Office of the Yale School of Medicine’ with written informed consent from all subjects. All subjects gave written informed consent in accordance with the Declaration of Helsinki. The protocol was approved by the ‘Yale Human Investigation Committee’, which is the Yale IRB (protocol number 1609018454).

## Author Contributions

ST, MH, EL, and RC contributed to the conception of the work. ST, MS, and VM-K designed the experiments. ST, KP, AV-R, VM-K, KN, and MS collected and analyzed the data. DS and MH helped with the implementation of the neurofeedback experiment, data collection, and analysis. All authors contributed to the interpretation of the results. ST drafted the manuscript. All authors revised it critically and provided final approval of the version to be published.

## Conflict of Interest Statement

MH has a patent application for neurofeedback in a different modality. The application is titled “Methods and systems for treating a subject using NIRS neurofeedback” (PCT/US2017/036532, filed June 8, 2017). The remaining authors declare that the research was conducted in the absence of any commercial or financial relationships that could be construed as a potential conflict of interest.
