# Supplementary material for: Insula as the Interface Between Body Awareness and Movement: A Neurofeedback-Guided Kinesthetic Motor Imagery Study in Parkinson’s Disease
Source: Front Hum Neurosci. 2018 Dec 7;12:496. doi: 10.3389/fnhum.2018.00496 (PMC6292989; doi:10.3389/fnhum.2018.00496)
Supplement: Supplementary file 1 [file Data_Sheet_1.pdf]

Supplementary Material

Title: Insula as the interface between body awareness and movement: A neurofeedback-guided kinesthetic motor imagery study in Parkinson's disease

Authors: *Sule Tinaz, Kiran Para, Ana Vives-Rodriguez, Valeria Martinez-Kaigi, Keerthana Nalamada, Mine Sezgin, Dustin Scheinost, Michelle Hampson, Elan D. Louis, R. Todd Constable

***Correspondence: Sule Tinaz: sule.tinaz@yale.edu**

Supplementary Data

Table S1. Demographic and clinical data of the first PD group (N = 10)

Gender	5 Male, 5 Female
Age	62.6 ± 10.8
Disease onset side	3 right, 7 left
Disease Duration (years)	6.1 ± 2.7
MDS-UPDRS total	53.9 ± 12.3
MDS-UPDRS III	33.3 ± 8.3
H&Y	2.1 ± 0.1
MoCA	26.8 ± 2.0

MDS-UPDRS: Movement Disorders Society Unified Parkinson's Disease Rating Scale, III: Part III motor exam.
H & Y: Hoehn and Yahr stage
MoCA: Montreal Cognitive Assessment battery

Self-evaluation questionnaires

To assess the levels of anxiety, depression, apathy, fatigue, and overall quality of life, subjects were administered the following questionnaires, respectively on visit day 1: Spielberger State-Trait Anxiety Inventory (STAI-S and STAI-T) (Spielberger et al., 1983), Beck Depression Inventory-II (BDI-II) (Beck et al., 1996), Starkstein Apathy Scale (Starkstein et al., 1992), Parkinson's Fatigue Scale (PFS) (Brown et al., 2005), and Parkinson's disease Quality of Life Questionnaire (PDQ-39) (Peto et al., 1995). Motor imagery skills were assessed using the Movement Imagery Questionnaire-3 (MIQ-3) (Williams et al., 2012). The MIQ-3 is an examiner-administered questionnaire that requires subjects to perform four complex movements, then imagine the movements focusing on different aspects of imagery including internal visual,

external visual, and kinesthetic. In the internal visual condition, they are instructed to "see" themselves performing the movements from an internal perspective through their own eyes and in the external visual condition to "watch" themselves perform the movements from an external perspective. In the kinesthetic condition, they are instructed to "feel" the movements. After each condition, subjects rate the difficulty of motor imagery on a Likert-type scale from 1 (very hard) to 7 (very easy).

Statistical analyses and results of the questionnaires completed by the second PD group:

Apathy scores were normally distributed ($p = 0.738$). We used the cutoff score of 14, where <14 is considered non-apathetic and ≥14 is considered apathetic (Starkstein et al., 1992). The mean apathy score of all subjects was 11.25. Three subjects were found to be apathetic and five were non-apathetic.

Based on the Beck Depression Inventory (BDI)-II scores depression severity is ranked as minimal (0-13), mild (14-19), moderate (20-28), and severe (29-63) (Beck et al., 1996). BDI scores were normally distributed ($p = 0.337$). The group mean BDI-II score was 7.63 and within the minimal depression range. Six subjects had minimal and two had mild depression.

Spielberger state anxiety scores were normally distributed in males ($p = 0.940$) and females ($p = 0.163$). The mean score in males (27.3) was not significantly different from the normative mean for males between the ages 50-69 (34.51 ± 10.34) ($p = 0.097$). The mean score in females (28.3) was also not significantly different from the normative mean for females between the ages 50-69 (32.20 ± 8.67) ($p = 0.115$) (Spielberger et al., 1983).

Spielberger trait anxiety scores were not normally distributed in males ($p = 0.037$) or females ($p = 0.007$). The median score in males (30) compared with the normative mean for males between the ages 50-69 (33.86 ± 8.86) was not significantly different (one-sample Wilcoxon nonparametric test, $p = 0.715$). The median score in females (29.5) was also not significantly different from the normative mean (31.79 ± 7.78) for females between the ages 50-69 (one-sample Wilcoxon nonparametric test, $p = 0.715$)) (Spielberger et al., 1983).

Parkinson's fatigue scale (PFS)-16 scores calculated using the Likert scale were normally distributed ($p = 0.831$). The mean PFS score was 2.32 ± 0.80 (raw scores divided by 16). In Brown et al., a cut-off score of 2.95 separated those who experienced fatigue from those who did not. A score equal to or greater than 3.30 was used to identify those who perceived fatigue as a problem (Brown et al., 2005). According to this classification, our PD cohort showed a trend for significant fatigue ($p = 0.061$), but fatigue was clearly not perceived as a problem ($p = 0.010$).

Parkinson's Disease Summary Index (PDSI) is a measure of the global impact of the disease on the wellbeing of the patient as measured by the Parkinson's disease quality of life questionnaire-39 (PDQ-39) (Jenkinson et al., 1997). It is calculated by summing the total normalized dimension scores and dividing it by eight which is the number of domains. PDSI scores, like PDQ-39 individual dimension scores, range from 0-100 where 0 denotes best quality of life and 100 denotes worst quality of life. PDSI scores were normally distributed ($p = 0.220$). The mean PDSI score of 13.79 ± 12.30 compared to the normative mean of 31.60 of PD patients with H & Y 2 (Jenkinson et al., 1997) was significantly lower ($p = 0.005$) indicating a significantly better quality of life in our group.

The average difficulty levels of motor imagery were rated 5.3 ± 1.3 before and 5.6 ± 0.7 after neurofeedback training for the internal visual, 5.7 ± 0.7 before and 5.8 ± 1.0 after neurofeedback training for the external visual, and 5.3 ± 1.0 before and 5.5 ± 0.7 after neurofeedback training for the kinesthetic condition. A score of 4 indicates neither easy nor hard,

5 somewhat easy, and 6 easy to perform. The difference in motor imagery difficulty ratings before and after neurofeedback training was not significant for the internal visual ($p = 0.371$), external visual ($p = 0.933$), and kinesthetic conditions ($p = 0.561$).

Heartbeat counting task

Outside the scanner

Subjects practiced the heartbeat counting task that is used frequently to assess interoceptive accuracy (Schandry, 1981) first outside the scanner. Subjects were asked to focus on their heartbeat and try to silently count it without manually checking their pulse. Simultaneously, a research associate manually recorded the subject's pulse. This task was performed five times using varying time windows of 20, 30, 40, 50, and 60 s presented in a random order using the *e-Prime* software (Schneider et al., 2012). At the end of each counting period subjects verbally reported their count.

The percent heartbeat counting accuracy during each block was calculated using the following formula:

(1 – (|actual heartbeat count – perceived heartbeat count|)/actual heartbeat count) x 100

The mean percent accuracy averaged across five blocks was 62 ± 29. There was no significant main effect of block duration ($F(4, 5) = 1.73$, $p = 0.252$) on accuracy.

The mean percent heartbeat counting accuracy in our PD group was similar to that reported by Ricciardi et al. (58 ± 20) (Ricciardi et al., 2016).

In the scanner

A rest period of 30 s was included in the beginning and end of each run to establish the baseline brain activity. The task instructions were projected onto a screen at the head of the scanner which subjects were able to view through a mirror mounted on the head coil that reflected the screen. The cue "get ready" was shown to signal the beginning of each heartbeat counting block. During the heartbeat counting block (30 s), subjects focused on their heartbeat and tried to count it silently without cheking their pulse. At the end of the 30 s block, subjects were cued to "stop" and presented four boxes for 6 s, each of which had a range of numbers displayed inside. Subjects responded by choosing the box with the range corresponding to their count using a four-button response pad. The aim of the response period was to keep the subjects engaged in the task. A rest period for 10 s followed the response. There were four heartbeat counting blocks per run and a total of three runs.

During the response period of the heartbeat counting task inside the scanner, all subjects correctly selected the box with the range that was consistent with their perceived heart rate during a 30 s-block in the behavioral practice session.

Figure S1. Examples of single subject right insula activations (green circles) ($N = 10$, $p < 0.001$, uncorrected; cluster size = 10 voxels). Color bars show the t values. There was individual variability in the activated portions of the insula extending from the mid to anterior dorsal parts and often including the frontal operculum during the heartbeat counting task.

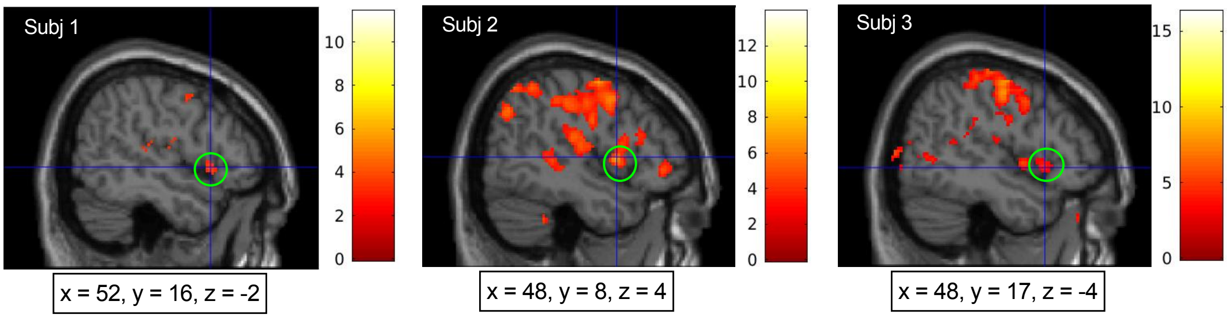

Table S2. Regions activated during heartbeat counting task

The x, y, z coordinates of peak activations based on the Montreal Neurological Institute standard template are listed. R: Right, L: Left, BA: Brodmann area, g: gyrus, SMA: Supplementary motor area, p. tri: Pars triangularis.

Region	BA	x	y	z	Z value
R primary motor cortex	4	58	0	10	4.73
L premotor cortex	6	-46	36	20	4.47
L SMA	6	-2	-2	72	4.44
L SMA/pre-SMA	6	-4	2	62	4.39
L mid insula	13/44	-42	6	6	4.33
R premotor cortex	6	54	-4	42	4.22
L premotor cortex	6	-48	-6	44	4.03
R premotor cortex	6	52	2	18	3.99
R visual association cortex	18	12	-80	-8	3.94
R mid insula	13	44	4	8	3.91
L inferior frontal g, p. tri	45	-50	24	4	3.86
L visual association cortex	18	-20	-78	-14	3.86
R supramarginal g	40	64	-36	38	3.70
L angular g	39	-48	-42	26	3.61

L frontal operculum	44	-38	12	10	3.60
R superior temporal g	22	62	-28	2	3.58
R primary visual cortex	17	8	-86	0	3.40
L primary motor cortex	4	-62	-8	14	3.36

Figure S2. **Activation during the response period of the heartbeat counting task.** Bilateral frontal operculum (A) and dorsal anterior cingulate cortex (B) (N =10, p < 0.001, uncorrected; cluster size = 10 voxels). Color bar shows the t values.

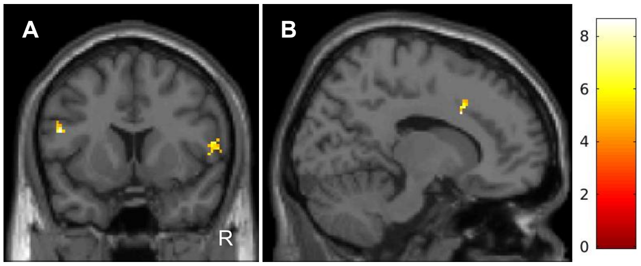

Table S3. **Peak coordinates of activations during the response period of the heartbeat counting task**

Region	BA	x	y	z	Z value
L frontal operculum	44	-50	10	20	4.37
R dorsal anterior cingulate	32	14	14	32	4.31
R frontal operculum	44	58	10	12	3.86

References

Beck A.T., Steer R.A., Brown G.K. (1996). Manual for the Beck Depression Inventory-II. San Antonio, TX: Psychological Corporation.

Brown R.G., Dittner A., Findley L., Wessely S.C. (2005). The Parkinson fatigue scale. *Parkinsonism Relat. Disord.* 11, 49–55. doi:10.1016/j.parkreldis.2004.07.007

Jenkinson C., Fitzpatrick R., Peto V., Greenhall R., Hyman N. (1997). The Parkinson's Disease Questionnaire (PDQ-39): development and validation of a Parkinson's disease summary index score. *Age Ageing* 26, 353–357

Peto V., Jenkinson C., Fitzpatrick R., Greenhall R. (1995). The development and validation of a short measure of functioning and well being for individuals with Parkinson's disease. *Qual. Life Res.* 4, 241–248.

Ricciardi L., Ferrazzano G., Demartini B., Morgante F., Erro R., Ganos C., et al. (2016). Know thyself: Exploring interoceptive sensitivity in Parkinson's disease. *J. Neurol. Sci.* (2016), 364, 110–115. doi:10.1016/j.jns.2016.03.019

Schandry R. (1981). Heart beat perception and emotional experience. *Psychophysiology* 18, 483-488.

Schneider W., Eschman A., Zuccolotto A. (2012). E-prime User's Guide. Pittsburgh: Psychology Software Tools, Inc.

Spielberger C.D., Gorsuch R.L., Lushene R.E., Vagg P.R., Jacobs G.A. (1983). Manual for the State-Trait Anxiety Inventory. Palo Alto, CA: Consulting Psychologists Press.

Starkstein S.E., Mayberg H.S., Preziosi T.J., Andrezejewski P., Leiguarda R., Robinson R.G. (1992). Reliability, validity, and clinical correlates of apathy in Parkinson's disease. *J. Neuropsychiatry Clin. Neurosci.* 4, 134–139. doi:10.1176/jnp.4.2.134

Williams S.E., Cumming J., Ntoumanis N., Nordin-Bates S.M., Ramsey R. (2012). Further validation and development of the motor imagery questionnaire. *J. Sport Exerc. Psychol*. 34, 621-646.
